# Assessing toxic work environments in Türkiye: adaptation and validation of the toxic work environment scale

**DOI:** 10.3389/fpubh.2026.1869949

**Published:** 2026-09-18

**Authors:** Elif Bilginoğlu, Uğur Yozgat

**Affiliations:** Faculty of Economics, Administrative and Social Sciences, İstanbul Nişantaşı University, İstanbul, Türkiye

**Keywords:** organizational behavior, scale adaptation, scale validation, toxic work environments, workplace toxicity

## Abstract

Toxic work environments have emerged as an important organizational issue due to their detrimental effects on employee well-being, job attitudes, and organizational effectiveness. Despite growing interest in workplace toxicity, there is a lack of validated instruments available for assessing toxic work environments in the Turkish context. Therefore, the primary aim of this study was to adapt and validate the Toxic Work Environment Scale for use in Türkiye. The study was conducted using a two-sample design involving 392 employees. The adaptation process followed established translation and back-translation procedures to ensure linguistic and conceptual equivalence. Exploratory factor analysis (EFA), confirmatory factor analysis (CFA), internal consistency analysis, and test–retest reliability assessment were performed to evaluate the psychometric properties of the scale. The findings supported a stable one-factor structure consistent with the original instrument. The scale demonstrated satisfactory internal consistency and temporal stability, indicating that it is a reliable and valid measure of toxic work environments in the Turkish context. The validated Turkish version of the scale provides researchers and practitioners with a useful tool for assessing workplace toxicity and examining its relationships with employee well-being, burnout, turnover intentions, absenteeism, and other organizational outcomes. The study contributes to the literature by providing a culturally adapted instrument that may facilitate future research on toxic work environments in Türkiye.

## 1 Introduction

Toxic work environments, defined as organizational climates characterized by persistent conflict, psychological unsafety, abusive supervision, and exclusionary practices, have become a critical subject in contemporary organizational behavior. These environments systematically erode employee well-being and organizational effectiveness (1–3). While considerable research has focused on the psychosocial consequences of toxicity in the workplace, its organizational and employee-related consequences remain an important area of investigation. Researchers in organizational behavior, occupational health, psychology, management, and human resource management have devoted decades to investigating workplace well-being (4). Grounded in foundational theories of organizational behavior, such as Edmondson’s (5) psychological safety, Schein’s (6) framework on organizational culture, and Lipman-Blumen’s (7) concept of toxic leadership, this study conceptualizes toxicity not merely as interpersonal dysfunction, but as a systemic failure embedded in culture, leadership, and emotional labor dynamics.

Empirical evidence demonstrates that toxic workplace behaviors, such as bullying, discrimination, despotic leadership, and cognitive distraction, significantly increase employee turnover, absenteeism, and presenteeism, leading to substantial productivity losses (8–10). According to McKinsey and Company (10) toxic culture is now the strongest global predictor of employee burnout and intent to leave, accounting for over 60% of negative workforce outcomes (11) (p. 8).

At the organizational level, toxic cultures are marked by broken feedback systems, favoritism, micromanagement, and emotional neglect—conditions that weaken internal cohesion and damage reputational capital (2). Additionally, such environments disproportionately affect vulnerable populations, including women, disabled employees, and older workers, thereby diminishing workforce participation and exacerbating socioeconomic inequalities (8). As a result, toxic workplace dynamics reduce the efficiency of human capital allocation and hinder inclusive growth. Therefore, this study aims to adapt and validate the *“Toxic Work Environment Scale”* (12) for use in the Turkish context. By providing a culturally and linguistically appropriate measurement tool, the research seeks to contribute to both theoretical and practical understandings of workplace toxicity in the Turkish context. The article is structured as follows: The next section presents a review of the relevant literature and theoretical foundations of toxic workplace cultures. This is followed by a detailed description of the methodology, including translation, adaptation, and validation processes. Subsequently, the results of exploratory and confirmatory factor analyses are reported. The final sections discuss the findings considering existing literature, outline the limitations, and propose policy and research implications.

Empirical evidence demonstrates that toxic workplace behaviors such as bullying, discrimination, despotic leadership, and cognitive distraction are associated with increased burnout, absenteeism, presenteeism, turnover intention, and reduced employee productivity (8–10). These findings highlight the importance of identifying and assessing toxic work environments in order to better understand their consequences for employees and organizations.

## 2 Theoretical background and literature review

The concept of toxic organizational culture has gained increased attention in recent years, especially as workplace well-being and psychological safety have become central themes in management research. A toxic work environment is typically characterized by persistent negative behaviors such as bullying, incivility, manipulation, exclusion, excessive micromanagement, and violations of personal boundaries. These environments foster stress, anxiety, and disengagement, thereby undermining individual health and organizational effectiveness (11, 13, 14).

The term *“toxic”* itself carries a strong metaphorical weight, originally rooted in the ancient Greek word *“toxon,”* meaning *“bow,”* and later Latinized as *“toxicus,”* referring to poisoned arrows. This etymology offers a symbolic understanding of toxic cultures: not the result of isolated incidents, but rather the accumulation of repeated, harmful interactions (15). Such cultures are not merely the product of unethical behavior by few individuals but are often embedded in the organization’s norms, leadership style, and feedback mechanisms (16, 17).

Scholars like Shain (3) define toxic workplaces as environments where abusive behaviors are normalized, either ignored or indirectly supported by management. These patterns include favoritism, inconsistent expectations, suppression of employee voice, and the erosion of mutual trust. When left unaddressed, these dynamics generate long-term psychological harm and foster cultures of fear, secrecy, and resignation (18, 19).

Wilde (19) further categorizes organizational toxicity into structural, emotional, cognitive, and behavioral dimensions. Structurally, toxic organizations are often marked by ambiguous hierarchies, rigid rules, and punitive systems. Emotionally, they promote environments where shame, guilt, and defensiveness are prevalent. Cognitively, these organizations stifle innovation and critical thinking through manipulation and suppression of dissent. Behaviorally, they manifest in passive-aggressive communication, scapegoating, and resistance to change.

The effects of such environments are severe. Studies show that employees in toxic cultures experience significantly higher levels of burnout, absenteeism, and emotional exhaustion (8, 10). Moreover, toxic behaviors disproportionately affect vulnerable populations, including women, older employees, and individuals with disabilities, thereby exacerbating existing inequalities in the workplace (11, 20).

Toxicity also affects organizational structures. It thrives in environments lacking clear values and consistent leadership. Where communication is unidirectional and feedback is not reciprocated, toxicity becomes ingrained. Over time, groupthink, manipulation, and exclusion become normalized; further damaging collaboration, performance, and employee morale (2, 21).

Given these risks, understanding the dimensions and indicators of toxic organizational culture is critical not only for psychological well-being but also for long-term organizational sustainability. Addressing toxic dynamics requires more than individual resilience; it calls for systemic change, inclusive leadership, and cultural transformation strategies (18, 22). In the Turkish context, the first comprehensive attempt to develop a measurement tool on organizational toxicity was undertaken by Kasalak (23), who constructed the *Perceived Organizational Toxicity Scale* specifically for higher education settings. While this instrument represents a valuable contribution to the national literature, it was developed within a particular institutional and cultural framework and thus has limited comparability with internationally established measures. By contrast, the scale introduced by Wang et al. (12) was designed to capture the multidimensional nature of organizational toxicity and has been applied across diverse organizational contexts. Therefore, without disregarding the significance of Kasalak’s (23) work, the present study aims to provide a more updated Turkish validation of Wang et al.’s (12) instrument in order to generate psychometric evidence that is not only reliable within the local context but also facilitates cross-cultural comparisons in the broader international literature.

## 3 Method

In accordance with Brislin’s (24) back-translation procedure, the original English version of the scale was translated into Turkish by a commission of four academicians proficient in English. Subsequently, the equivalence between the Turkish and English versions was examined to ensure semantic and conceptual consistency. Later, the Turkish form was examined in terms of meaning and grammar by three academicians in Turkish language and necessary corrections were made, and the Turkish form was obtained for trial. In the next step, the Turkish trial form was applied to eight doctoral and seven master’s degree students who took a leadership course, and minor changes were made in line with the opinions of these students. To ensure participant protection, all individuals were provided with an informed consent form prior to participation and voluntarily agreed to complete the surveys.

### 3.1 Sample

The data for the scale validation study were collected in two separate phases. The first sample comprised 258 employees, primarily drawn from the healthcare and education sectors, who were surveyed between April 12 and April 27, 2025. In June and July 2025, the questionnaire was administered twice to a second sample of 400 employees from the same occupational backgrounds, of whom 134 completed both waves. In total, the study sample consisted of 392 employees.

### 3.2 Toxic workplace environment scale

The Toxic Workplace Environment Scale, developed by Wang et al. (12), comprises seven items (see Appendix). Participants rated each item using a six-point Likert scale ranging from 1 (Strongly Disagree) to 6 (Strongly Agree). Based on the authors’ analysis, all items were retained in the scale without modification.

### 3.3 Scale validation process

According to Hinkin (25), the scale evaluation phase—recommended as a critical component of scale development—encompasses the assessment of validity and reliability for the refined instrument. In this study, factor analyses, internal consistency, and test–retest reliability analyses were conducted. The first sample was utilized for factor analysis and internal consistency assessment, while the second sample served to evaluate test–retest reliability. In social sciences, scales are generally expected to exhibit moderate to high correlations over time, as constructs shaped by socialization processes tend to influence individuals for extended periods and are assumed to remain stable rather than situationally variable. Therefore, scale’s time consistency was examined.

Although the original scale items explicitly refer to *“supervisor”* behaviors, the Turkish adaptation intentionally reframed the item stems at the workplace level (e.g., *“in my work environment”*) rather than attributing behaviors solely to a single individual. This decision was guided by the conceptual scope of the construct measured by the scale, namely toxic workplace environment, which refers to enduring organizational climates and normalized interaction patterns rather than isolated dyadic leader–subordinate relationships. Directly translating the term *“supervisor”* into Turkish (*“amirim”*) risked narrowing the construct toward abusive supervision and leadership-specific behaviors, thereby underrepresenting the broader environmental and cultural dimensions of workplace toxicity.

Accordingly, the adaptation employed passive and environment-focused phrasing while preserving the original behavioral content, temporal qualifiers, and evaluative intensity of each item. This approach ensured maximum semantic equivalence with the source instrument while enhancing construct validity by aligning item wording with the theoretical definition of toxic work environments as systemic and contextually embedded phenomena. Consistent with established cross-cultural adaptation guidelines, the modification was limited to the grammatical framing of agency and did not alter item meaning or measurement intent, allowing the scale to retain its psychometric integrity while achieving greater conceptual coherence in the Turkish context.

### 3.4 Findings

In the first stage of the study, confirmatory factor analysis (CFA) was conducted, and the scale items loaded onto a single factor, consistent with the original study. In the second stage, exploratory factor analysis (EFA) was performed to examine the underlying factor structure and item distribution. Prior to EFA, sampling adequacy was assessed using the Kaiser–Meyer–Olkin (KMO) measure, and the appropriateness of item correlations was evaluated with Bartlett’s test of sphericity. The results indicated that the KMO value (0.852 > 0.60) was excellent for factor analysis, and Bartlett’s test [χ^2^ = 852.905; df = 21; *p* < 0.001] was significant, confirming the suitability of the data for factor analysis (26).

Given the absence of a prior validation study in the Turkish context and the possibility that cultural factors may influence the scale’s underlying structure, Principal Component Analysis (PCA) was performed to identify the factor structure, and the Direct Oblimin rotation method was employed to account for correlations among factors (27, 28). Decisions regarding item retention were based on factor loadings and cross-loadings. Specifically, items were retained if their primary loading exceeded 0.60, and in cases of cross-loading, the difference between factor loadings was greater than 0.30 (28). If an item is removed from the analysis, the analysis was repeated until the exploratory factor analysis was conducted from the beginning and the above criteria were met. None of the items need to be removed. The results showed that the scale has a one-factor structure which explains 57.24% of the total variance. According to the EFA structure matrix, one factor consists of all seven items and has an acceptable internal consistency coefficient (Table 1). At this stage, confirmatory factor analysis (CFA) was conducted using the one-factor model identified through exploratory factor analysis (EFA), following the recommendations of Worthington and Whittaker (28). Although some fit indices were borderline, the proposed model demonstrated an acceptable fit for the combined sample (*N* = 392), GFI = 0.917, TLI = 0.897, CFI = 0.896, SRMR = 0.052.

**Table 1:** Toxic workplace environment scale (Turkish form).

Items	Mean	Std. Dev.	Factor loadings
TWE-5	1.69	1.094	0.840
TWE-1	2.08	1.270	0.803
TWE-6	1.72	1.073	0.780
TWE-4	1.77	1.186	0.779
TWE-3	2.37	1.167	0.731
TWE-7	1.78	1.045	0.713
TWE-2	2.72	1.318	0.631
Eigenvalue			4.007
Total variance explained			57.244
Cronbach’s alpha			0.871

To assess test–retest reliability, data collected from 134 participants at two time points, June and July 2025, were analyzed. This analysis aimed to evaluate the stability of the construct over time by examining correlations between measurements at T1 and T2. Item-level correlations ranged from 0.379 to 0.714, confirming the temporal stability of the construct (see Table 2).

**Table 2:** Toxic workplace environment scale (Turkish form): test–retest validity.

Items	Mean (T_1_)	Mean (T_2_)	Correlation
TWE-1	2.31	2.22	0.663^**^
TWE-2	2.84	2.63	0.379^**^
TWE-3	2.45	2.53	0.560^**^
TWE-4	1.98	1.91	0.696^**^
TWE-5	1.82	1.95	0.714^**^
TWE-6	1.90	2.04	0.678^**^
TWE-7	1.83	2.07	0.628^**^

**p<0.01.

## 4 Conclusion

This study highlights toxic organizational culture as a significant organizational challenge. Unless workplace toxicity is identified and addressed through appropriate organizational interventions, its adverse consequences for employee well-being, burnout, absenteeism, turnover intentions, and organizational functioning may continue to affect workplaces. The findings underscore the importance of identifying and addressing workplace toxicity through evidence-based organizational interventions, supportive leadership practices, and the promotion of psychologically safe work environments.

Organizational and occupational health research increasingly requires validating Wang et al.’s (12) *“Toxic Work Environment Scale”* in Türkiye, offering both a methodological innovation and a practical diagnostic tool. By comparing local data with broader datasets, these tools contribute to the development of internationally recognized theories of employee well-being and organizational health (29). The validated Turkish version of the Toxic Work Environment Scale provides a reliable tool for advancing research and practice aimed at understanding and reducing workplace toxicity. Future studies may use this validated instrument to examine the relationships between toxic work environments and employee well-being, burnout, absenteeism, turnover intention, organizational performance, psychological safety, and other workplace outcomes.

## Data Availability

The raw data supporting the conclusions of this article will be made available by the authors, without undue reservation.

## Appendix

**Table A1:** Toxic workplace environment scale (Turkish form).

TWE-1 Çalıştığım iş ortamında, benimle açık sözlü olmaya çalışılarak sık sık bazı müstehcen şakalar paylaşılmaktadır.
TWE-2 Çalıştığım iş ortamında, fiziksel görünüşüm sık sık takdir edilmektedir.
TWE-3 Çalıştığım iş ortamında, bana yetkinlik seviyeme uygun olmayan işler verilmektedir.
TWE-4 Çalıştığım iş ortamında, sık sık kişisel ve cinsel hayatım hakkında konuşulmaya çalışılmaktadır.
TWE-5 Çalıştığım iş ortamında, selam verdiğimde selamıma cevap verilmediği durumlar yaşanmaktadır.
TWE-6 Çalıştığım iş ortamında, bana karşı mesafeli davranılmaktadır.
TWE-7 Çalıştığım iş ortamında, benimle topluluk içinde kaba bir şekilde konuşulmaktadır.
